# Supplementary material for: Factors associated with the use of dental services in older adults in Peru
Source: PLoS One. 2025 Feb 12;20(2):e0316651. doi: 10.1371/journal.pone.0316651 (PMC11819527; doi:10.1371/journal.pone.0316651)
Supplement: Attachment — Submitted filename: PLOS One review.docx [file pone.0316651.s001.docx]

This study uses data on older adults in Peru to examine potential predictors of dental service use (termed as access to dental services). There are several major and minor issues that inhibit my enthusiasm for this study and must be clarified or addressed before moving forward.

**Major comments**

The gap this study is addressing is not clear to me. The authors state that previous work has identified these factors as being predictive of dental service utilization. How is this study building on this and contributing something new? The authors should provide more information and support for how this study offers new insights.

There is very little information given on what the authors did for “quality control” of the data, yet it is noted as a strength of the study. More detail needs to be given on what quality control entailed.

Please provide more detail about the ENDES. Is this meant to be a nationally-representative sample? How are the data for the ENDES collected? Are data collected annually, and are there linkages across years if someone is surveyed multiple times (or are these anonymized repeated cross-sectional data)?

I am still unclear on how the outcome variable (access to dental services) was defined. It seems to be parameterized dichotomously, however the sentence describing it does not make clear the window of time for a “recent” visit; is there a threshold by which the most recent visit would not indicate someone has access to dental services? For example, if someone had a dental visit in July of 2018 and no other visits, are they considered to have access to dental services? This versus someone who has more frequent visits throughout a year may be an important distinction. More clarity is needed on how the outcome variable was defined.

Related to the above, is there any information on the nature of the dental visits? Someone may have a single visit and reasonable considered as having access to dental services if they are just receiving a routine cleaning, however someone else may have a single visit and be in need of more significant dental work and be unable to access it due to financial constraints; would this person also be counted as having access to dental services?

It would be helpful to more clearly differentiate the measures described in the methods. Specifically, the authors could consider creating subsections with headings for “outcome”, “predictors” and “covariates for adjustment” so it is clear to the reader which measures are of interest for their potential predictiveness and which are only for adjustment.

Why were some covariates included to adjust the models but results not reported, while others were? Specifically, the authors mention education as a covariate but also include its regression estimates, while language and department are not. Language is examined using a bar chart instead, but not talked about in the discussion section of the manuscript. Why wasn’t language discussed?

**Minor comments**

How are the levels of the wealth index defined? Is wealth based solely on income, or are there other factors considered (assets, properties, etc.)? A one-sentence description of this would be helpful.

Including the variable codes seems extraneous; it takes up a lot of space and makes it more difficult to read through the methods. I would remove them.

The authors should, in clarifying their definition of the outcome, make clear that they are (or seem to be) using dental service utilization as a proxy for access (note that access and utilization are closely tied but not exactly the same).

Please explain why some factors (i.e., age, region, and marital status) were not included in the multivariable model.

Please change “multivariate” to “multivariable” as the former describes models with multiple outcomes, whereas the latter describes models with multiple covariates.
