# Supplementary material for: Factors associated with the use of dental services in older adults in Peru
Source: PLoS One. 2025 Feb 12;20(2):e0316651. doi: 10.1371/journal.pone.0316651 (PMC11819527; doi:10.1371/journal.pone.0316651)
Supplement: Attachment — Submitted filename: Response to the Reviewers.doc [file pone.0316651.s002.doc]

| Reviewers' Comments | Response from the Authors |
| --- | --- |
| ****REVIEWER 1.**** It is unclear what gap this study is addressing. The authors state that previous studies have identified these factors as predictors of dental service utilization. To what extent does this study build on that and provide something new? The authors should provide more information and support on how this study contributes new insights. | The reviewer's comment was taken into account and promptly addressed. |
| ****REVIEWER 1.**** Very little information is provided regarding what the authors did for "data quality control," yet it is highlighted as a strength of the study. More details are needed on what the quality control process entailed. | The reviewer's comment was taken into account and promptly addressed. |
| ****REVIEWER 1.**** Provide more details about ENDES. Is it designed to be a nationally representative sample? How are the data collected in ENDES? Are the data collected annually, and are there links across years if the same individual is surveyed multiple times, or are they repeated cross-sectional and anonymous data? | The reviewer's comment was taken into account and promptly addressed. |
| ****REVIEWER 1.**** I still do not fully understand how the outcome variable (access to dental services) was defined. It seems to be parameterized dichotomously; however, the description does not clarify the time period for a "recent" visit. Is there a threshold beyond which the most recent visit would not indicate access to dental services? For instance, if someone had a dental visit in July 2018 and no further visits, would they be considered to have access to dental services? This contrasts with someone who has more frequent visits over a year, which could represent an important distinction. Greater clarity is needed on how the outcome variable was defined. | The reviewer's comment was taken into account and promptly addressed. |
| ****REVIEWER 1.**** In relation to the above, is there any information about the nature of the dental visits? For example, one person might have a single visit and reasonably be considered to have access to dental services if they are only receiving a routine cleaning. However, another individual might have a single visit and require more extensive dental work but be unable to access it due to financial limitations. Would this person also be considered as having access to dental services? | Dear reviewer, the mentioned limitation has been added to the discussion. |
| ****REVIEWER 1.**** It would be helpful to differentiate the measures described in the methods more clearly. Specifically, the authors could consider creating subsections with headings such as "Outcome," "Predictors," and "Covariates for Adjustment" to make it clear to the reader which measures are of interest for their predictive potential and which are only used for adjustment. | The reviewer's comment was taken into account and promptly addressed. |
| ****REVIEWER 1.**** Why were some covariates included to adjust the models but their results were not reported, while others were? Specifically, the authors mention education as a covariate and also include its regression estimates, whereas language and department are not reported. Language is analyzed through a bar chart, but it is not mentioned in the discussion section of the manuscript. Why was language not addressed? | The exclusion of certain covariates, such as language, from the multivariable model and the discussion was primarily due to collinearity with other variables included in the analysis, such as geographic region and wealth index, which more broadly capture structural inequalities related to access to dental services. While language may play an important role in specific local contexts, its effect was not independent of these variables in preliminary analyses. Therefore, a methodological decision was made to prioritize variables that better represent the general sociodemographic dynamics of the country. However, we acknowledge that this decision may have limited the exploration of certain specific linguistic barriers, and we have highlighted this limitation in the discussion section as an area to address in future research. |
| ****REVIEWER 1.**** How are the levels of the wealth index defined? Does wealth rely solely on income, or are other factors such as assets, properties, etc., taken into account? A concise description of this would be helpful. | The reviewer's comment was taken into account and promptly addressed. |
| ****REVIEWER 1.**** Including the variable codes seems unnecessary, takes up significant space, and makes the methods section harder to read. I would recommend removing them. | The reviewer's comment was taken into account and promptly addressed. |
| ****REVIEWER 1.**** The authors should clarify their definition of the outcome and explicitly state that they are (or appear to be) using the utilization of dental services as a proxy for access. Please note that while access and utilization are closely related, they are not exactly the same. | The reviewer's comment was taken into account and promptly addressed. |
| ****REVIEWER 1.**** Please explain why some factors (e.g., age, region, and marital status) were not included in the multivariable model. | The exclusion of certain covariates, such as language, from the multivariable model and the discussion was primarily due to collinearity with other variables included in the analysis, such as geographic region and wealth index, which more comprehensively capture structural inequalities related to access to dental services. While language may play an important role in specific local contexts, its effect was not independent of these variables in preliminary analyses. Therefore, a methodological decision was made to prioritize variables that better represent the broader sociodemographic dynamics of the country. Nevertheless, we acknowledge that this decision may have limited the exploration of specific linguistic barriers, and we have highlighted this limitation in the discussion section as an area to be addressed in future research. |
| ****REVIEWER 1.**** Replace "multivariate" with "multivariable," as the former refers to models with multiple outcomes, while the latter describes models with multiple covariates. | The reviewer's comment was taken into account and promptly addressed. |
| ****REVIEWER 2.**** The manuscript title needs to be revised to reflect that the study determined the factors associated with the utilization of dental services. | The reviewer's comment was taken into account and promptly addressed. |
| ****REVIEWER 2.**** The definition of dental service utilization (last dental visit <2 years or ≥2 years) requires an explanation and justification. Why was the 2-year threshold chosen? Is visiting the dentist at least once every 2 years the recommended frequency in Peru? It is unlikely that this threshold is comparable to research conducted in many other countries where the recommended frequency is higher, such as at least once or even twice per year. | The reviewer's comment was taken into account and promptly addressed. |
| ****REVIEWER 2.**** Given the large sample size, it seems feasible to consider more than two categories for dental service utilization, such as within the last year, 1–2 years, and more than 2 years. At the very least, more descriptive summaries of dental service utilization could be provided. However, was dental service utilization perhaps only asked as a dichotomous response (<2 years and ≥2 years)? In any case, it is not evident that the authors have given much thought to the definition of the outcome variable. | We acknowledge that the large sample size could have allowed for a more detailed categorization of dental service utilization, such as visits within the last year, between 1 and 2 years, and more than 2 years. However, the ENDES survey collected this information through a structured question with a dichotomous response (<2 years or ≥2 years), limiting the possibility of a more specific classification. Additionally, this definition was chosen due to the lack of clear national recommendations regarding the ideal frequency of dental visits, aiming to strike a balance between capturing recent access and ensuring consistency with previous local studies. Nevertheless, we recognize that this choice may simplify some of the complex dynamics of access, and we have included this limitation in the discussion, emphasizing the need for future studies to use more detailed data collection tools for a more precise characterization of dental service utilization. |
| ****REVIEWER 2.**** The selection of potential factors associated with dental service utilization requires justification. Were these the only characteristics collected in the survey? Were measures regarding the need for dental care or the reasons for the last dental visit not included? | Dear Reviewer,  We appreciate your thoughtful comments and take this opportunity to provide further clarification:  The variables selected for this study, including sex, age, educational level, type of residence, health insurance affiliation, wealth index, self-perceived ethnicity, and physical limitations, are relevant as they represent well-documented sociodemographic and economic determinants of access to healthcare. These variables help identify structural and contextual inequities that directly influence dental service utilization among older adults in Peru. For instance, educational level and wealth index are often associated with the ability to prioritize and afford dental care, while type of residence and geographic region reflect physical and logistical barriers, particularly in rural and remote areas.  However, other potentially relevant variables could not be included due to limitations in the data available from ENDES. These include information on the perceived need for dental care, specific reasons for not utilizing dental services, detailed cultural or linguistic barriers, and the quality or type of services received. The absence of such data restricts the ability to capture more complex and detailed dynamics of access to dental services. This limitation underscores the importance of future studies incorporating broader and more specific data collection tools to address these additional dimensions and enhance understanding of the barriers and facilitators to accessing oral healthcare. |
| ****REVIEWER 2.**** The description of the regression method is somewhat vague: "a robust variance estimate was used to account for potential distortions in the data." I would recommend describing the method as "crude and adjusted prevalence ratios were calculated using a modified Poisson regression with robust standard error estimates to account for overdispersion." | The reviewer’s comment was taken into account, and the description was revised as follows: Crude and adjusted prevalence ratios were calculated using a modified Poisson regression with robust standard error estimates to account for overdispersion in the data. |
| ****REVIEWER 2.**** In the title of Table 2, clearly indicate that it reports on factors associated with the lack of access to dental services. Additionally, Table 2 should include the overall statistical significance of each variable, as is done in Table 1. | The reviewer's comment was taken into account and promptly addressed. |
| ****REVIEWER 2.**** It is somewhat disconcerting that the authors do not comment on the difference between the crude prevalence rate and the adjusted prevalence rate by type of residence (a prevalence 255% higher for lack of access to dental care versus a prevalence only 5% higher for lack of access to dental care). Should the reader assume that the apparent lack of access to dental care in rural areas compared to urban areas is due to other factors, such as education and wealth? That is, is dental care access similar in rural and urban areas, but rural residents cannot afford dental care or do not consider it important? A more likely explanation is that there are strong correlations between factors beyond just the region of residence, which the authors should consider. | The reviewer's comment was taken into account and promptly addressed. |
| ****REVIEWER 2.**** What about the interactions between factors? It is not mentioned whether the authors accounted for interactions between variables. Once again, the analysis appears to be underdeveloped. | The reviewer's comment was taken into account and promptly addressed. |
