# Supplementary material for: Factors associated with the use of dental services in older adults in Peru
Source: PLoS One. 2025 Feb 12;20(2):e0316651. doi: 10.1371/journal.pone.0316651 (PMC11819527; doi:10.1371/journal.pone.0316651)
Supplement: Attachment — Submitted filename: ACT-Response to the Reviewers.doc [file pone.0316651.s003.doc]

| COMMENTS FROM THE ACADEMIC EDITOR AND REVIEWER 2 | Response from the Authors |
| --- | --- |
| However, the Editor and Reviewer 2 do not see the authors address the interactions between variables in their recent revision. If the authors believe that the reviewer's comment was taken into account and promptly addressed, please list the page number and line number of the updated content for the Editor's consideration. The Editor would suggest the authors add a paragraph in the Discussion Section to discuss the influecnce from the interactions between variables as shown in Table 2. For example, why was the aPR of the ethnoracial, education, residence type, and wealthy index variables become less significant after the adjustment with other variables? This likely resulted from the interactions among variables. Additionaly, why was the region variable not included in the multivariave model as shown in Table 2? In Lines 180-183 you specified that Adjustment covariates included... Geographical region... Please explain and/or discuss these. | We sincerely appreciate the comments provided, as we did not fully understand the request during the initial review. These comments have been instrumental in enhancing the clarity and depth of the manuscript. In response, we have added a paragraph in the Discussion section that examines how the interaction and overlap of determining factors explain the reduced significance of certain variables after adjustment. Additionally, we have clarified that the exclusion of the geographic region variable from the multivariable model was justified due to collinearity issues, as other included variables more accurately capture the structural inequalities affecting access to dental services. These revisions aim to provide a more thorough and rigorous analysis of the results.  The modification can be viewed on page 19, lines 388–402. |
